# Towards Personalized Precision Oncology: A Feasibility Study of NGS-Based Variant Analysis of FFPE CRC Samples in a Chilean Public Health System Laboratory

**DOI:** 10.3390/cimb47080599

**Published:** 2025-07-30

**Authors:** Eduardo Durán-Jara, Iván Ponce, Marcelo Rojas-Herrera, Jessica Toro, Paulo Covarrubias, Evelin González, Natalia T. Santis-Alay, Mario E. Soto-Marchant, Katherine Marcelain, Bárbara Parra, Jorge Fernández

**Affiliations:** 1 Subdepartamento de Genómica y Genética Molecular, Laboratorio Nacional Biomédico y de Referencia, Instituto de Salud Pública de Chile, Av. Marathon 1000, Ñuñoa, Santiago 7780050, Chile; eduran@ispch.cl (E.D.-J.); iponce@ispch.cl (I.P.); mrojash@ispch.cl (M.R.-H.); pcovarrubias@ispch.cl (P.C.); bparra@ispch.cl (B.P.); 2 Departamento de Oncología Básico Clínico, Laboratorio de Genómica del Cáncer, Facultad de Medicina, Universidad de Chile, Av. Independencia 1027, Independencia, Santiago 8380453, Chile; jessicatoro@uchile.cl (J.T.); evefeliu@gmail.com (E.G.); kmarcelain@uchile.cl (K.M.); 3 Centro para la Prevención y el Control del Cáncer (CECAN), Facultad de Medicina, Universidad de Chile, Av. Independencia 1027, Independencia, Santiago 8380453, Chile; 4 Departamento Agencia Nacional de Dispositivos Médicos, Instituto de Salud Pública de Chile, Av. Marathon 1000, Ñuñoa, Santiago 7780050, Chile; natalia.santis@uchile.cl (N.T.S.-A.); mesoto2@uc.cl (M.E.S.-M.); 5 Departamento de Tecnología Médica, Facultad de Medicina, Universidad de Chile, Santiago 8380453, Chile; 6 Escuela de Tecnología Médica, Facultad de Salud y Odontología, Universidad Diego Portales, Santiago 8340263, Chile

**Keywords:** precision medicine, NGS, actionable variants, cancer biomarkers, public health

## Abstract

Massively parallel or next-generation sequencing (NGS) has enabled the genetic characterization of cancer patients, allowing the identification of somatic and germline variants associated with their diagnosis, tumor classification, and therapy response. Despite its benefits, NGS testing is not yet available in the Chilean public health system, rendering it both costly and time-consuming for patients and clinicians. Using a retrospective cohort of 67 formalin-fixed, paraffin-embedded (FFPE) colorectal cancer (CRC) samples, we aimed to implement the identification, annotation, and prioritization of relevant actionable tumor somatic variants in our laboratory, as part of the public health system. We compared two different library preparation methodologies (amplicon-based and capture-based) and different bioinformatics pipelines for sequencing analysis to assess advantages and disadvantages of each one. We obtained 80.5% concordance between actionable variants detected in our analysis and those obtained in the Cancer Genomics Laboratory from the Universidad de Chile (62 out of 77 variants), a validated laboratory for this methodology. Notably, 98.4% (61 out of 62) of variants detected previously by the validated laboratory were also identified in our analysis. Then, comparing the hybridization capture-based library preparation methodology with the amplicon-based strategy, we found ~94% concordance between identified actionable variants across the 15 shared genes, analyzed by the TumorSec^TM^ bioinformatics pipeline, developed by the Cancer Genomics Laboratory. Our results demonstrate that it is entirely viable to implement an NGS-based analysis of actionable variant identification and prioritization in cancer samples in our laboratory, being part of the Chilean public health system and paving the way to improve the access to such analyses. Considering the economic realities of most Latin American countries, using a small NGS panel, such as TumorSec^TM^, focused on relevant variants of the Chilean and Latin American population is a cost-effective approach to extensive global NGS panels. Furthermore, the incorporation of automated bioinformatics analysis in this streamlined assay holds the potential of facilitating the implementation of precision medicine in this geographic region, which aims to greatly support personalized treatment of cancer patients in Chile.

## 1. Introduction

According to the latest report by the Global Cancer Observatory (GLOBOCAN) and the International Agency for Research on Cancer (IARC), there were close to 20 million new cases, and approximately 9.7 million deaths from cancer in 2022 (including non-melanoma skin cancers) [1]. Worldwide, the most prevalent types of cancer were breast (BC), colorectal (CRC), trachea–bronchus and lung (TBLC), prostate (PC), thyroid, and gastric cancer (GC) [1]. On the other hand, the most fatal cancers in 2022 included LC, CRC, liver, BC, and GC [1]. Chile recorded 31,440 cancer-related deaths and 59,876 new cancer diagnoses in 2022. By gender, 16,897 men and 14,543 women lost their lives to cancer, with the deadliest types being PC, GC, TBLC, CRC, and liver cancer in men, and BC, CRC, TBLC, GC, and pancreatic cancer in women. Projections suggest a 26.5% increase in cancer incidence and a 30.4% rise in mortality in Chile by 2030 compared to 2022 (https://gco.iarc.fr/; accessed on 1 March 2025). This underscores the significance of cancer as a critical public health issue nationally and globally, highlighting the need for innovative and improved diagnostic and therapeutic strategies. In 2020, a new National Health Strategy was developed in Chile, incorporating indicators from the National Cancer Plan for 2018–2028. This strategy is focused on the improvement of cancer diagnosis and treatment through next-generation sequencing (NGS) analysis. The advent of NGS has significantly improved cancer care globally by providing a comprehensive tumor genetic profile. These technologies have enhanced the understanding of the variability of responses to antineoplastic therapies among individuals with the same type of tumor.

The increasing sophistication of oncology therapy and clinical trials is largely attributed to the targeting of genetic biomarkers in tumors, often investigated via NGS (fda.gov) (https://www.cancer.gov; accessed on 1 March 2025) [2,3,4]. The growing body of research in this area has been fundamental to the development of precision medicine. This approach is rooted in the understanding that patients are distinct and may respond differently to treatment, even when facing the same cancer. This variability underscores the significance of precision medicine and the necessity of employing its analytical strategies to personalize treatments, ultimately aiming for improved patient survival and quality of life. A significant barrier to advancing precision oncology in Chile and Latin America is limited access to NGS tumor testing for identifying actionable variants. This is largely due to the substantial costs associated with implementing and sustaining these technologies, particularly within public healthcare systems. Consequently, testing is performed in private laboratories and/or samples are sent abroad, resulting in higher costs and prolonged turnaround times, restricting treatment options for patients and their families. A cost-effective strategy for adopting NGS in these regions involves targeted sequencing of specific genomic regions of interest (ROIs) to detect clinically relevant genetic variants [5]. Target enrichment of these ROIs during library preparation can be achieved through PCR amplification using specific primer panels (amplicon-based methods) or hybridization with sequence-specific probes followed by separation of the captured sequences (hybridization capture-based methods) [5].

In this study, we aimed to implement the identification, annotation, and prioritization analyses of relevant actionable genetic somatic variants using a retrospective cohort of 67 formalin-fixed, paraffin-embedded (FFPE) CRC samples in the laboratory of Genomics and Molecular Genetics from the Chilean Public Health Institute, as an integral part of the public health system. We compared two different library preparation approaches: an amplicon-based sequencing assay (Illumina AmpliSeq v2 Hotspot Panel) that is designed to target hotspot regions of 50 genes and a hybridization capture-based library preparation method, TumorSec^TM^, from the Universidad de Chile. This assay uses the KAPA HyperPlus kit (Roche) and SeqCap EZ target Capture System (Roche), with a custom panel of probes originally designed to analyze 25 genes relevant in Chile and Latin America for approved oncological drugs. TumorSec^TM^ includes a bioinformatics pipeline designed for Latin and Hispanic populations [6]. For sequencing data analysis and genomic variant annotation, the bioinformatics pipelines TumorSec^TM^ and two commercial platforms (Franklin by Genoox and CLC platform from QIAGEN) were used. The results obtained allowed us to identify the pros and cons of each methodology in terms of concordance, turnaround time, analysis time, and cost.

We retrospectively verified our results with those obtained previously by the Cancer Genomics Laboratory (CGL) at the Universidad de Chile, which is the group that validated the TumorSec^TM^ pipeline. Using 28 previously analyzed CRC samples, we obtained 80.5% concordance between our results and those obtained previously at the CGL, using the same library preparation (hybridization capture-based) and bioinformatics analysis approaches (namely TumorSec^TM^), but with different sequencers (Miseq (original study) vs. Nextseq (our analysis)). Notably, 98.4% of variants detected in the original analysis were also identified by our analysis. Next, comparing the capture-based library preparation method against the amplicon-based strategy, we found ~94% concordance between actionable variants identified in the 15 shared genes analyzed by the TumorSec^TM^ bioinformatics pipeline. Similar concordance results in terms of the identified variants were obtained using the CLC bioinformatics commercial platform and the Franklin database compared with in-house-developed TumorSec^TM^ analysis, demonstrating its accuracy and robustness.

The results of our study indicate that the feasibility of implementing an NGS-based analysis for the detection and prioritization of actionable variants in cancer samples is viable in our laboratory, thereby offering a pathway to enhance access to these crucial analyses within the Chilean public health system. Recognizing the economic constraints prevalent in much of Latin America, employing a targeted NGS panel focusing on relevant variants for the Chilean and Latin American population presents a cost-efficient strategy compared to extensive global panels. Furthermore, the incorporation of automated bioinformatics analysis (like TumorSec^TM^) into this optimized assay promises to expedite the implementation of precision medicine in this region, with the goal of significantly improving the diagnosis and personalized treatment of cancer patients in Chile.

## 2. Materials and Methods

### 2.1. FFPE Tumor Specimens

Formalin-fixed, paraffin-embedded (FFPE) CRC tumor samples were obtained from the Tissue and Fluid Biobank at the Universidad de Chile (BTUCH). The biobank provided authorization to use samples that already had informed consent and are approved for their use in research. A total of 67 FFPE CRC specimens were analyzed using the TumorSec^TM^ and AmpliSeq v2 Cancer Hotspot strategies. Of the samples, 43.3% (29 out of 67) were from female patients and 50.7% (34 out of 67) were from male patients. The patient’s sex is unknown for 4 samples. The average age of patients at diagnosis was 61.2 years, ranging between 33 and 86 years. Histologically, 74.6% of samples have a tubular carcinoma classification and 71.6% of samples have a TNM classification of T3-T4. The collected FFPE samples contained at least 20% tumor tissue, validated by an expert pathologist at BTUCH. Supplementary Table S1 illustrates the FFPE samples’ characteristics.

### 2.2. Control Samples

A reference pathological standard DNA sample from Horizon Discovery (HD200 FFPE somatic; Cambridge, UK) was used. This FFPE sample, which has specific mutations at known variant allelic frequencies (VAFs), was used as a positive control to estimate the sensitivity and precision of variant calling. Additionally, to evaluate assay specificity, one apparently healthy control sample from the Coriell Institute for Medical Research (NJ, USA) was used (NA01990).

### 2.3. DNA Extraction, Quantification, and Quality Control

FFPE tissue DNA was extracted using the GeneJet FFPE DNA Purification Kit and RecoverAll^TM^ Total Nucleic Acid Isolation Kit (Invitrogen, Thermo Fisher Scientific, Carlsbad, CA, USA), following the manufacturer’s instructions. Purified DNA was quantified using the Qubit^TM^ double-stranded DNA (dsDNA) High Sensitivity (HS) Assay (Invitrogen). DNA purity was assessed by measuring the 260/280 nm absorbance ratio. For FFPE samples, fragment length and degradation were assessed using Bioanalyzer 2100 or TapeStation D5000 (Agilent; Santa Clara, CA, USA). DNA ranged from >1000 bp to 200 bp. Samples with <200 bp are not recommended for processing with the TumorSec^TM^ workflow.

### 2.4. Library Preparation and Target Enrichment

Library preparation using the hybridization capture-based methodology was performed, following the manufacturer’s instructions, with the KAPA HyperPlus library preparation (Roche; Basel, Switzerland) and SeqCap EZ target Capture System (Roche) kits, with minor technical modifications, as described by M. Salvo et al. [6]. The hybridization probes used for target captures target 25 genes, containing more than 300 targets therapeutically relevant in Chile and Latin America population. A measure of 250 ng of DNA extracted from FFPE samples were used as input. Libraries were purified using Ampure XP magnetic beads (Beckman Coulter; Brea, CA, USA). The size and integrity of the libraries were evaluated using TapeStation D5000 and quantified using Qubit dsDNA HS Assay. For target enrichment, the prepared DNA libraries (1200 ng total mass) were captured by custom hybridization probes (Roche NimbleGen SeqCap EZ. Roche) and each capture reaction was performed with 4–5 individual sample libraries. Captured libraries were assessed for concentration and size distribution to determine molarity again, using TapeStation D5000. For the amplicon-based methodology, genomic libraries were prepared with the AmpliSeq v2 Cancer Hotspot commercial library preparation kit (Illumina Inc., San Diego, CA, USA), according to the manufacturers’ instructions, for subsequent amplicon-based sequencing. For this protocol, 50 ng of DNA from each sample was used. Finally, for control sample preparation, 100 ng DNA was used for the capture-based strategy and 10 ng DNA was used for the amplicon-based method. The lists of genes analyzed by TumorSec^TM^ and AmpliSeq v2 Cancer Hotspot, including the shared genes, are shown in Supplementary Table S2.

### 2.5. Next-Generation Sequencing

Libraries were diluted to 2 nM and processed according to the manufacturers’ instructions (Illumina, San Diego, CA, USA). These libraries were denatured with 0.2 N NaOH and then diluted to 1.2 pM to be loaded. The samples, along with the different controls, were sequenced on a NextSeq^TM^ 550 device (Illumina) with 2 × 150 paired-end sequencing, with a 300 cycle V2 sequencing kit (Illumina). PhiX control was included in each run at a final concentration of 5%. A maximum of 39 samples were loaded.

### 2.6. Bioinformatics Analyses

We used the TumorSec^TM^ bioinformatics pipeline to analyze NGS data generated by both library preparation strategies (capture-based and amplicon-based), replacing the BED file, which contains target genomic coordinates, in each case. The TumorSec^TM^ pipeline was developed by researchers from the Universidad de Chile and is exhaustively described by M. Salvo et al. [6]. The bioinformatics pipeline and tutorial are available in the GitHub repository called Pipeline-TumorSec (https://github.com/u-genoma/Pipeline-TumorSec, accessed on 1 January 2024). Additionally, the CLC Workbench (Version 24.0.2; QIAGEN) software was also used to analyze NGS data to identify genetic somatic variants, and to compare the results compared to the TumorSec^TM^ pipeline. The Targeted Amplicon Sequencing workflow was used for this analysis. Filtering parameters were customized to compare the results with those obtained using the TumorSec^TM^ pipeline.

### 2.7. Variants Prioritization

**Figure 1:**
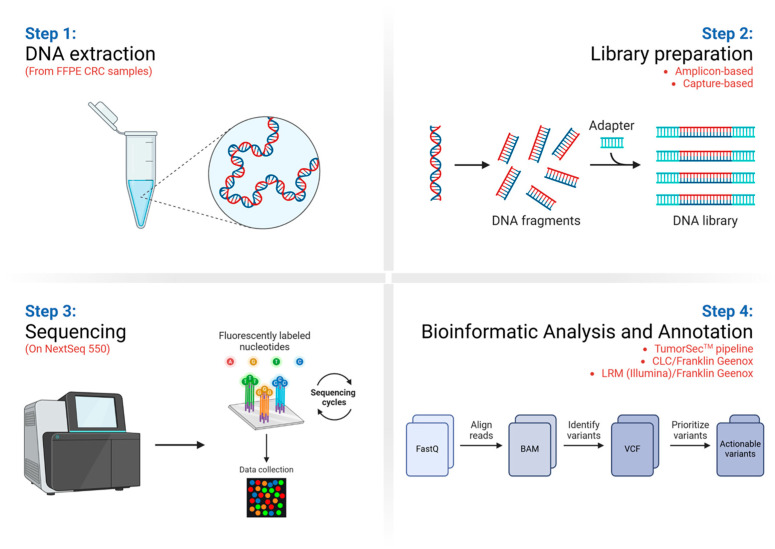
General scheme of the NGS workflow. (Step 1—(**upper left**)) Genomic DNA extraction from FFPE CRC samples was performed. Briefly, the GeneJet FFPE DNA Purification Kit and RecoverAll^TM^ Total Nucleic Acid Isolation was used (Invitrogen), following the manufacturer’s instructions. Purified DNA was quantified using the Qubit^TM^ dsDNA HS Assay (Invitrogen). DNA purity (260/280 ratio) was measured. Fragment length and degradation were assessed using Bioanalyzer 2100 or TapeStation D5000 equipment (Agilent). The obtained DNA should have a fragment size from >1000 bp to 200 bp. (Step 2—(**upper right**)) Target DNA libraries were made using amplicon- or capture-based strategies (Ampliseq^TM^ and TumorSec^TM^, respectively). The detailed protocols are mentioned in Section 2.4. (Step 3—(**lower left**)) After library preparation, DNA-targeting sequencing was performed in a Nextseq^TM^ 550 (Section 2.5). (Step 4—(**lower right**)) Finally, bioinformatics analyses and variant annotation were performed using different strategies (for instance, TumorSec^TM^, CLC workbench (QIAGEN), Franklin (Genoox). Image created in BioRender.

The TumorSec^TM^ pipeline was also used to identify therapeutically actionable genetic variants derived from both library preparation strategies. The pipeline automatically filters variants against several databases such as the Catalogue of Somatic Mutations in Cancer (COSMIC), dbSNP, and CLINVAR, as well as population variant databases (PVDs) like GnomAD, ESP6500, ExAC, and 1000 Genomes. Identification of the actionable variants was performed using the Cancer Genome Interpreter database. All filters and parameters are available in [6] and in the GitHub repository called Pipeline-TumorSec (https://github.com/u-genoma/Pipeline-TumorSec; accessed on 1 March 2025). We applied a cut-off of at least 5% variant allele frequency (VAF) and at least 200X target depth. Finally, we also analyzed the unfiltered variant data obtained with both library preparation and sequencing protocols (Variant Call Format or VCF files) with the Franklin commercial database (Genoox). These VCF files were generated from the raw FASTQ files, either with the CLC Workbench (QIAGEN) software or with the DNA Amplicon Analysis Module (Illumina), and added to the Local Run Manager Software (Illumina) installed in the NextSeq^TM^ 550 sequencer. Figure 1 shows a summary of the study workflow, including (1) DNA extraction from FFPE samples, (2) DNA library preparation, (3) DNA sequencing by NGS, and (4) bioinformatics analyses and variant annotation.

## 3. Results

### 3.1. Resequencing Analysis of FFPE CRC Samples Using the TumorSec^TM^ Pipeline

**Figure 2:**
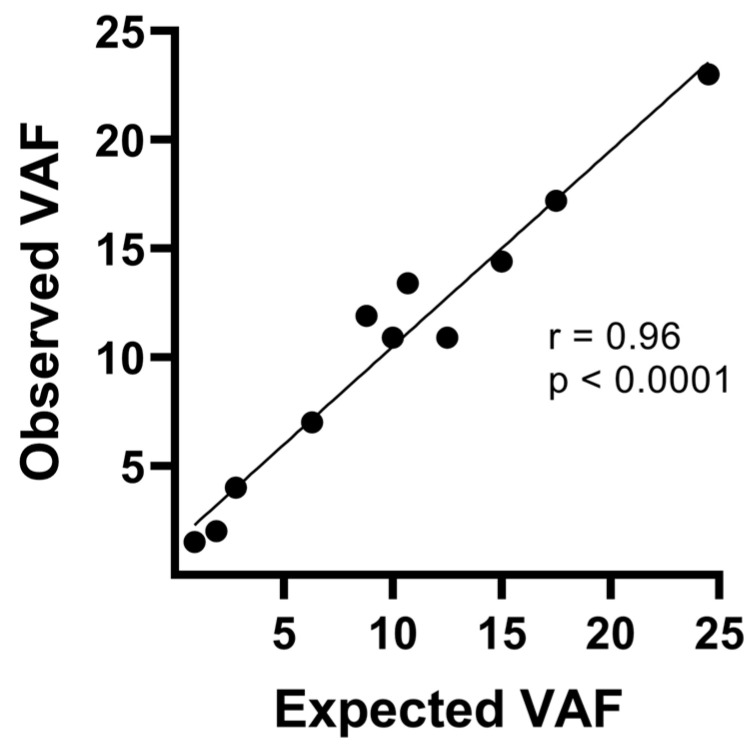
Verification of performance and analytical sensitivity of the TumorSec^TM^ panel. Correlation between expected allele frequencies (*x*-axis) for informed variants in a commercial standard FFPE control sample and those observed by the assay (*y*-axis). The HD200 FFPE Horizon Discovery sample was used.

In order to begin the implementation of the TumorSec^TM^ NGS methodology in our laboratory, we used the commercial reference standard (HD200; Horizon) to evaluate panel performance and accuracy. The HD200 Horizon FFPE reference standard is a highly characterized, biologically relevant quality control material, commonly used to assess the performance of NGS assays that detect somatic mutations with different VAFs. We detected all 11 variants reported in the HD200 reference and obtained a correlation coefficient of 0.96 (*p* < 0.0001) between expected and reported variant allele frequencies (Figure 2). Expected and reported VAFs ranged between 0.9 and 24.5%, demonstrating the high sensitivity of the procedure and the validity of the results obtained. Furthermore, we did not detect any pathological variant in an apparently healthy control sample (NA01990; Coriell), confirming the specificity of the assay.

Then, to obtain the first insights for the implementation of actionable variant detection in our laboratory, we compared our results against those obtained previously at the CGL group, using the same CRC patients’ samples and methodologies but sequenced on a NextSeq^TM^ 550 machine instead of a MiSeq. The TumorSec^TM^ pipeline considers a panel of 25 genes containing more than 300 targets therapeutically relevant and prevalent in Chile and the Latin America population. Of a total of 67 FFPE CRC samples, 28 were analyzed in both laboratories. An 80.5% concordance between our results and those in CGL was obtained, using the same library preparation (capture-based) and bioinformatics analysis approaches. However, we identified 77 variants, while the CGL reported 63. As we increased sequencing depth using NextSeq^TM^ 550, we also detected additional variants in some samples. For instance, low frequency variants (VAF~5%) such as *ARID1A* c.G2959A, (VAF 0.06; sample CC001), *PDGFRA* c.G1676T (VAF 0.06; sample CC014) or *PIK3CA* c.G1070T (VAF 0.05; sample CC016) were only detected by our analysis. Notably, after prioritization, 98.4% of actionable variants (62 out of 63 variants) detected in the validated laboratory were also detected by our analysis. The complete list of variants identified by both studies is presented in Table 1.

Importantly, NextSeq^TM^ 550 sequencing of CRC FFPE samples showed a high rate of duplicated reads. However, average uniformity was >90% in all samples analyzed and more than 91% of targeted regions had >300X depth, metrics that validate sequencing findings. These and other sequencing quality metrics are shown in (Supplementary Table S3).

### 3.2. Comparison Between Library Preparation Strategies in the Identification of Actionable Variants

**Figure 3:**
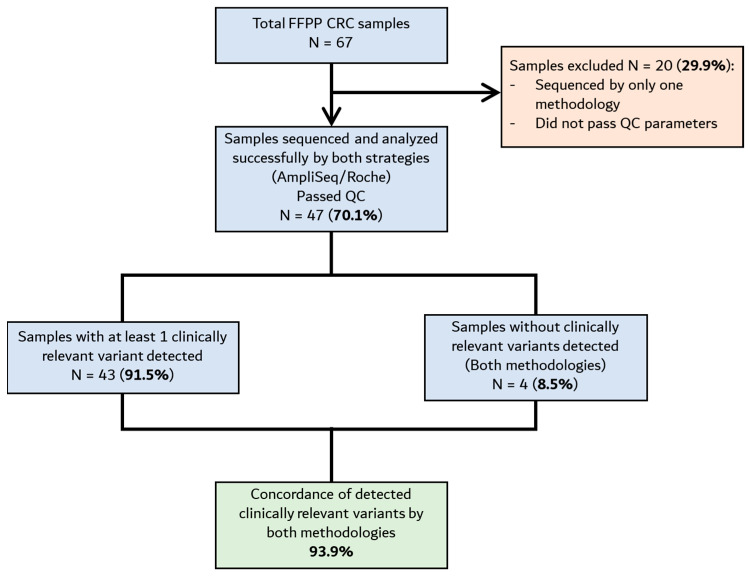
Flowchart depicting the inclusion/exclusion criteria for FFPE CRC samples for the analysis of variant identification by NGS. Of the 67 initial FFPE samples obtained, 47 were suitable for analysis by both methodologies (amplicon- and capture-based).

After analyzing FFPE CRC samples with the TumorSec^TM^ pipeline (including the capture-based library preparation and the bioinformatics), we aimed to compare the performance of two different library preparation kits, including the capture-based strategy used with TumorSec^TM^ for the identification and prioritization of relevant genetic variants. Therefore, we also used a commercial amplicon-based method (AmpliSeq v2 Cancer Hotspot, Illumina) as a comparator. The FFPE CRC samples were processed, and library preparation was performed with both kits. NGS results were analyzed with the same bioinformatics pipeline, i.e., TumorSec^TM^, to identify and annotate clinically relevant variants in genes shared by both strategies (Supplementary Table S2). Of the 67 initial FFPE CRC samples, 20 did not pass quality control filters/parameters in terms of DNA integrity, quantity, or bioinformatics quality control (QC). Thus, 47 samples were completely sequenced and analyzed by both strategies (Figure 3).

**Figure 4:**
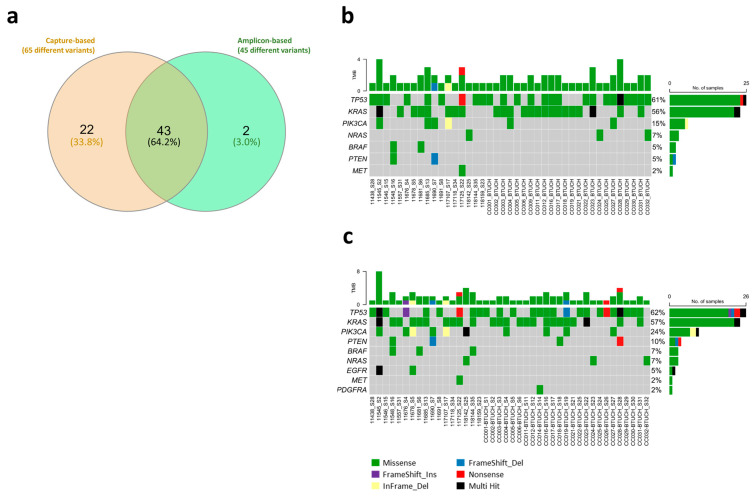
Characterization of actionable variants identified by amplicon- and capture-based methodologies across 47 FFPE CRC samples and within the 15 shared genes by both strategies. (**a**) In total, 43 out of 45 variants detected by the commercial amplicon-based method were also detected by the capture-based method. On the other hand, 22 actionable variants were only detected using the capture-based strategy. (**b**,**c**) Onclopots showing actionable variants detected in each sample and each gene by either (**b**) amplicon-based or (**c**) capture-based methodologies. Mutations observed are classified as somatic (somatic, possible somatic, and possible somatic novel) that produce a change in the protein, with VAF >= 5% and MAF (Minor allele frequency) <= 1% in PVDs (Population Variant Databases). The Venn diagram (**a**) was constructed using the web-tool designed by Heberle et al. [7]. Oncoplots (**b**,**c**) were made using the same TumorSec^TM^ bioinformatics pipeline.

The results show a high concordance between the two library preparation kits. Considering individual unique variants, 43 out of 45 variants (95.6%) detected by the commercial amplicon-based method were also detected by the capture-based method (Figure 4a). On the other hand, although they are variants that should be identified by both strategies, several (22 out of 65 variants; 33.8%) actionable variants were only detected using the capture-based strategy (Figure 4a). Coincidentally, most of these variants showed a VAF close to the filtration threshold (5%). The depicted oncoplots show the individual actionable variants detected in each sample by gene (Figure 4b,c). As expected, most of the actionable variants detected in our CRC samples are in the *TP53* and *KRAS* genes, and corresponded to missense variants. Additionally, we obtained a ~94% concordance of annotated clinically relevant variants when processing the samples with these two different library preparation kits (capture- vs. amplicon-based strategies) (Table 2). Overall, these results suggest that the capture-based methodology, which includes in-house-designed probes and the bioinformatics TumorSec^TM^ [6] pipeline, could potentially be used together with other library preparation strategies (i.e., predesigned/predefined) to prioritize relevant and therapeutically actionable genetic variants all in one software/pipeline, which is open-access/free and could considerably and significantly reduce the time consumed by bioinformatics analysis.

- Total prioritized variants detected by amplicon-based and capture-based approaches (TumorSec^TM^ bioinformatics): 61 (93.8%).
- Amplicon-based-only detected variants (TumorSec^TM^ bioinformatics): 4 (6.2%).
- Total prioritized variants detected by capture-based and amplicon-based approach (TumorSec^TM^ bioinformatics): 61 (73.5%).
- Capture-based-only detected variants: 22 (26.5%).

Finally, we aimed to test the results obtained with the amplicon-based library preparation methodology (Ampliseq Cancer Hotspot v2 panel), analyzed either with the in-house-developed bioinformatics pipeline TumorSec^TM^ or with the commercially available CLC workbench software v24.0.2 (to obtain the VCF and BAM files), using the Targeted Amplicon Sequencing workflow (QIAGEN). Additionally, we also used the BED and VCF files to annotate variants using the Franklin platform (Genoox). Filters and quality control values were adjusted in CLC to be comparable with the TumorSec^TM^ bioinformatics pipeline (described in the Section 2). Having made this adjustment, and after sample QC filtration and annotation-prioritization using Franklin, we obtained a 96.7% concordance between the variants identified by TumorSec^TM^ and CLC/Franklin analyses, meaning that 59 out of the 61 variants identified by CLC analysis were also identified by the TumorSec^TM^ bioinformatics pipeline (Table 3—3rd and 4th columns). Importantly, samples where no variants were found by CLC/Franklin also coincided when analyzed by TumorSec^TM^. Similarly, and as expected, after generating the VCF files using the DNA Amplicon Analysis Module from the Local Run Manager Software (LRM), and following annotation and prioritization using the Franklin database (LRM/Franklin), a 100% concordance was achieved between the two softwares (Table 3—4th and 5th columns) (Table 3), demonstrating an excellent robustness between the different pipelines in identifying, prioritizing and annotating clinically relevant variants.

- There was 96.7% concordance between CLC/Franklin vs. TumorSec^TM^ annotated variants (59 out of 61 variants—3rd and 4th columns; amplicon-based library preparation);
- There was 100% concordance between CLC/Franklin vs. LRM/Franklin (4th and 5th columns; amplicon-based library preparation);
- There was 87.9% concordance between amplicon-based library preparation + Franklin variants annotation vs. capture-based library preparation + TumorSec^TM^ annotation analysis (80 out of 91 variants).
- ✔ indicate the variant was detected by that particular pipeline
- **✕** indicate the variant wasn’t detected by that particular pipeline

Supplementary Table S4 summarizes the findings of this study, including actionable vari-ants identified in each sample, as well as their VAF and OncoKB classification (oncogenicity) and actionability. Variants that are not classified by OncoKB were classified by the TumorSec^TM^ algorithm. For instance, *PDGFRA* c.1676G>T is a non-synonymous variant reported in lung cancer (COSMIC ID 21163438; Genomic mutation identifier (COSV) COSV57273507) which is predicted to be a somatic tumor-driver mutation. However, most actionable variants are or are predicted to be oncogenic/likely oncogenic according to OncoKB database, with level 1/2 therapy response to an FDA-approved drug, R1 evidence to be a biomarker predictive of resistance to an FDA-approved drug in this indication of clinical significance, and FDA level 2 evidence of clinical significance.

## 4. Discussion

Over the past decade, oncology has undergone substantial changes in the management of cancer, increasing the focus on precision medicine [8]. Many clinical studies have focused on the genetic analysis of tumors in patients around the world; many of those studies, analyzed by NGS, demonstrated that the response to the newly available treatments is largely determined by specific genetic mutations in tumor cells [9,10]. However, limited access to this strategy is still a significant health concern, mostly in terms of cost, and especially in Latin American and other developing countries. Here, we have verified the results obtained and validated in the Cancer Genomics Laboratory (CGL) using the methods described by M. Salvo et al. [6], after retrospectively identifying clinically relevant variants in FFPE CRC samples, and have implemented the methodology to identify and annotate these variants in our laboratory, as an integral part of the Chilean public health system.

First, we obtained highly concordant results comparing ours versus those obtained by the CGL (as the validated laboratory for this methodology). We achieved 80.5% concordance (62 out of 77 variants), using the same library preparation and bioinformatics analyses approaches (capture-based approach + TumorSec^TM^ bioinformatics) (Table 1); only one variant in the *TSC2* gene (c.5383C>T) was detected in the CGL and not in our study. As we increased sequencing depth using the NextSeq^TM^ 550 equipment, we also detected additional variants in some samples. For instance, low-frequency variants (VAF ~5%), such as *ARID1A* c.G2959A (sample CC001; VAF 0.06), *PDGFRA* c.G1676T (sample CC014; VAF 0.06) or *PIK3CA* c.G1070T (sample CC016; VAF 0.05), were detected only by our analysis. Notably, 62 out of 63 variants (98.4%) detected by the CGL group were also detected by our analysis (Table 1). Second, we obtained ~94% concordance of annotated clinically relevant variants, processing samples with two different library preparation kits based on different methods (capture- vs. amplicon-based strategies), and analyzing the obtained sequencing data with two different (yet, with the same filters) bioinformatics pipelines, including the in-house-developed TumorSec^TM^ pipeline [6] (Table 2). This means that actionable variants detected in samples processed by the commercial AmpliSeq Cancer Hotspot Panel v2 panel (amplicon-based) were also detected using the KAPA/Roche library preparation strategy (capture-based). It is worth noting that, as mentioned in the Section 2, the analyses here were performed only on those variants that were common between the two library preparation kits (70 variants in total). Similarly, to the first results, there were variants only detected using the KAPA/Roche/TumorSec^TM^ strategy (capture-based; 21 variants). This could be explained because they showed a VAF close to the threshold (5%). Also, there are differences in the regions covered by each kit. For example, the *KRAS* gene is fully covered by the capture-based strategy (TumorSec^TM^) (covering all exons/full gene), while amplicon-based methodology includes primers to target just three hotspot regions. Finally, it has been described that hybridization capture-based methods are less prone to fail in the detection of mutations than the amplicon-based methods [5,11]. Considering that the TumorSec^TM^ pipeline is designed to analyze clinically relevant variants in the Latin American population, the great performance and concordance with the results obtained with a known and widely distributed commercially available kit (namely Ampliseq) suggests that TumorSec^TM^ could be used to prioritize and annotate actionable variants all in one in-house-developed, open-access software/pipeline. Using this pipeline could also reduce the time and, most probably, the cost of this kind of analysis. Finally, we also compared the use of two additional different commercially available platforms to annotate actionable variants identified in FFPE CRC samples processed with the AmpliSeq Cancer Hotspot v2 library preparation method, namely CLC (QIAGEN) and Franklin (Genoox). We obtained 96.7% concordance between AmpliSeq/CLC/Franklin versus AmpliSeq/TumorSec^TM^/Franklin, which means that 59 out of 61 actionable variants were identified and annotated by CLC and by TumorSec^TM^ bioinformatics (Table 3—3rd and 4th columns). Notably, and as expected, we obtained 100% concordance between AmpliSeq/CLC/Franklin vs. AmpliSeq/LRM/Franklin, as they use the same input files (BAM, BED and VCF files) to perform the annotation (Table 3—4th and 5th columns).

With cancer incidence rates increasing, there is an urgent need to implement NGS technology and genetic variant detection in Latin American public health institutions. Moreover, there is evidence of its success in other regions, improving early diagnosis and patient outcomes. For instance, studies have shown that NGS can aid in the detection of actionable mutations in cancers, allowing for targeted therapies that can significantly prolong patient survival [12,13,14,15]. Although NGS testing encompasses multiple types of assays, another interesting review summarized the current evidence on the clinical impact of using NGS tests to guide management of patients with cancer in the United States. Analyzing more than 30 publications where progression free survival (PFS) and overall survival (OS) were evaluated, they saw that PFS or OS were significantly longer among patients who were studied by NGS testing and matched to targeted treatment in 11 and 16 publications across tumor types, respectively, suggesting that NGS-informed treatment could improve patient survival [16]. Moreover, considering cost-effectiveness and accessibility, the European Society for Medical Oncology (ESMO) has recently updated their recommendations regarding NGS testing in advanced cancer patients in routine practice; expanded their 2020 recommendations to BC and rare tumors such as gastrointestinal stromal tumors, sarcoma, thyroid cancer, and cancer of an unknown primary source; and also recommended tumor NGS for detecting tumor-agnostic alterations, where matched therapies are accessible [14].

Although the advantages of NGS are evident, the adoption of this technology within Latin American public health systems is significantly hampered by several interconnected challenges. The considerable expense of NGS tests and technologies often creates a barrier to access for patients, impacting both low- and middle-income populations. A significant number of public health institutions still lack the fundamental physical and technological infrastructure, along with the skilled human resources, required to establish comprehensive or even targeted genomic testing programs. Moreover, the absence of dedicated funding from local governments for NGS technologies and assays, combined with a lack of reimbursement mechanisms for these crucial analyses within the public health system, frequently leads to reliance on private laboratories. This inevitably increases costs and severely restricts accessibility for patients. Furthermore, there is an urgent need to improve and/or create public health policies, including on topics such as when to perform a tumor NGS analysis, how to perform the analysis, a targeted or comprehensive study, what the available financial coverage is for patients, among others, to regulate the implementation of tumor NGS studies [17,18,19]. Thus, strong collaborations between governments, policymakers, universities, healthcare institutions, patient associations, and international organizations are crucial to secure funding and establishing NGS technology. Adequate training for healthcare professionals in genomics is also essential, including technical skills as well as training in genetic counseling, which is a significant gap in Chile and in the Latin American population. Ensuring that oncologists and genetic counselors are equipped with the knowledge to interpret NGS data and implement findings into treatment plans is vital for successful integration into clinical practice [20]. Finally, public health campaigns to raise awareness about genetic testing and its benefits may encourage more patients to seek such diagnostic options. These variables and others are exhaustively discussed in a recent article by Vacarezza, C. et al., which provides a detailed view of the current scene and future improvements currently needed in the Chilean NGS oncologic sphere [21].

Regarding the economic aspect, a recent review by Tan and collaborators pointed out that, although NGS is an effective tool for identifying mutations in cancer patients, more rigorous cost-effectiveness studies are needed to determine whether NGS can improve cancer patient outcomes [22]. For instance, a Canadian study evaluated the total costs of testing, including the costs of delaying medical care, associated with NGS versus single-gene testing strategies among patients with newly diagnosed LC, from the perspective of public financing. Among 1,000,000 hypothetical adults with Canadian public health insurance (382 with LC), the proportion of patients who tested positive for a genomic alteration eligible for an approved targeted therapy was 38.0% for NGS and 26.1% for single-gene strategies. The estimated median time to appropriate initiation of targeted therapy was also shorter for those who underwent NGS testing (5.1 vs. 9.2 weeks), and the costs associated with delayed care was lower for patients tested with NGS vs. single-gene strategies (3480 vs. 5632 Canadian dollars). The authors concluded that NGS can identify more patients with a mutation, deliver a shorter time to the appropriate initiation of targeted therapy, and lower total testing costs compared with single-gene strategies [23].

The lack of these kinds of studies and the economic constraints in most Latin American countries, including Chile, have led to cancer genomic testing being primarily concentrated in North America, Europe, and Asian countries. Moreover, well-established cancer databases such as The Cancer Genome Atlas (TCGA) and population databases generally do not include Latin American populations or ethnic groups; there is a significant challenge in accurately identifying frequent variants in these populations [6]. For instance, the percentage of those with American Indian ancestry among cancer patients across all cohorts in TCGA is around 4%. To increase the accuracy of somatic variant identification in our population, further local efforts are needed. It is hoped that this issue could be addressed with the Chilean Cancer Plan and the National Cancer Law, prioritizing funding for cancer research, particularly in the field of NGS technology [20,21].

In this study, we used two library preparation kits, each utilizing a different technique (Roche/hybrid capture-based vs. Illumina/amplicon-based), to evaluate which one could be more suitable for identifying and annotating actionable variants in our context. We also aimed to assess turnaround time and the time spent on bioinformatics analyses. We obtained ~94% concordance in the detection of annotated actionable variants using both kits, meaning that 94% (62 out of 66 variants) of the amplicon-based-method-detected variants were also detected by the capture-based library preparation strategy and TumorSec™ bioinformatics pipeline (Table 2). On the other hand, ~75% of capture-based method detected variants were also detected when using the amplicon-based library preparation kit (62 out 83). This means that 21 variants (which are evaluated by both kits) were detected and annotated only by the TumorSec^TM^ pipeline (Table 2). As mentioned in the Section 3, most of these 21 variants showed a VAF close to the filtration threshold (5%), which suggests that a capture-based library preparation method (such as Roche/TumorSec^TM^), may offer better sensitivity for detecting low-frequency variants. Enhancing variant detection sensitivity (from 5% to 1% for example) would be a key strategy to improve this methodology. Both methodologies have pros and cons according to their own characteristics. For instance, it is known that amplicon-based library preparation methods are more rapid (one full day of laboratory work in our experience) and require minimum amounts of DNA input. Also, they usually have more simplified workflows and perform well with more degraded samples. However, they could detect more false positives and discordant results because of PCR-associated biases [24]. In addition, amplicon-based strategies are often limited in the number of genomic targets that can be assessed in one panel (by the complexity in primers design PCR setting), affecting their scalability. These problems can be overcome by using different panels to analyze a larger number of targets. For example, the AmpliSeq Cancer Hotspot Panel v2 does not target mutations in *BRCA1/2* genes, but it can be complemented with specific panels, such as the AmpliSeq BRCA panel. Notably, the TurmorSec^TM^ capture-based method includes specific probes to capture *BRCA1/2* entire genes, thus it can identify somatic (and germline) variants in those genes [6]. Also, hybridization capture-based strategies are less likely to miss mutations and perform better with respect to sequencing complexity and uniformity of coverage and the probe panels can be escalated more easily than the primer panels in amplicon-based methodologies [11,24,25,26]. On the other hand, capture-based strategies need more initial, less damaged DNA input, require more technical expertise, and generally have a time-intensive workflow (2–3 days in our case). To address these challenges, new kits like the KAPA HyperPETE Somatic Tissue DNA Workflow (Roche) have been designed to reduce the total time required for the library’s preparation (1 day). All these factors must be considered when choosing one methodology or another and depend on the specific necessities, goals, and funds of each laboratory.

The TumorSec^TM^ pipeline, which is a hybrid capture-based methodology, includes in-house-designed probes to evaluate relevant genes and variants, focusing on the Latin American population, particularly the Chilean population (333 total variants present in 25 genes) [6]. This fact is both a strength and a limitation at the same time. However, as TumorSec^TM^ uses customized probes, there is always space to include new ones as knowledge regarding cancer-related variants emerges. As this method is mainly focused on the identification of small single-nucleotide variants (SNVs) and small insertion-deletions (indels), it has the limitation that it does not detect fusions and large structural variants which could be important and relevant to study for some cancer patients. We are currently working on the implementation of the detection of these variants. It is important to mention that most of the genes are fully evaluated by the Roche/TumorSec^TM^ pipeline, unlike the commercially available, pre-designed AmpliSeq Cancer Hotspot v2 by Illumina, which is an important strength. Finally, an important limitation of this study is the limited number of retrospective samples analyzed, and the fact that they only correspond to FFPE CRC samples. Nevertheless, the TumorSec^TM^ assay was previously validated in CRC and ovarian, breast, gastric, and gallbladder cancer. Additionally, as formalin fixation is known to create “artifact” genetic variants, it is desirable to implement this methodology when working with fresh biopsies. As this is difficult in the Chile (and most probably in the entirety of Latin America), this should be the long-term goal of private and public health NGS laboratories. We are currently working on the verification of this method in our laboratory, diversifying the cancer type of the samples analyzed, such as BC and LC samples. Also, short- and long-term follow up analyses of prospective tumor samples would give us valuable information associating Chilean cancer patients’ genetic variation and clinical survival, providing the possibility to use this information to improve public health policies and decision making. However, this is a very important advancement for public health system laboratories, as we could verify and correctly implement this methodology.

## 5. Conclusions

In conclusion, we have implemented the identification, annotation, and prioritization of actionable variants in FFPE tumor samples by NGS in our laboratory, as part of the Chilean public healthcare system. This kind of analysis could be offered to cancer patients in the public system to obtain better insights into their disease, which could greatly help in their clinical management, including by providing a tool for better diagnosis, therapeutic decision-making, and inclusion in clinical trials [17,27]. We hope that this study will pave the way to promote NGS diagnosis in the public health system and to be the first step in the implementation of other analyses, not only in the oncology field, but also in other genetic diseases.

## Figures and Tables

**Table 1:** Actionable variants identified by TumorSec^TM^ bioinformatics pipeline after sequencing FFPE CRC samples, and comparison with those previously identified at the Cancer Genomics Laboratory (CGL), as a validated laboratory for this methodology.

Sample	Variants Previously Found (CGL)	Variants Found in This Study
CC001	*TSC2* c.5185C>T *TP53* c.524G>A -	*TSC2* c.5185C>T *TP53* c.524G>A ***ARID1A* c.2959G>**A
CC002	*KRAS* c.34G>T	*KRAS* c.34G>T
CC003	*KRAS* c.35G>T *TP53* c.524G>A	*KRAS* c.35G>T *TP53* c.524G>A
CC004	*KRAS* c.183A>C *PIK3CA* c.1634A>G	*KRAS* c.183A>C *PIK3CA* c.1634A>G
CC005	*TP53* c.455C>T	*TP53* c.455C>T
CC006	*KRAS* c.35G>A	*KRAS* c.35G>A
CC008	*KRAS* c.346A>C *TP53* c.524G>A *PIK3CA* c.3140A>G *BRAF* c.1741A>T	*KRAS* c.346A>C *TP53* c.524G>A *PIK3CA* c.3140A>G *BRAF* c.1741A>T
CC009	*BRCA2* c.2588delA *ARID1A* c.3977delC *PIK3CA* c.3140A>G	*BRCA2* c.2588delA *ARID1A* c.3977delC *PIK3CA* c.3140A>G
CC011	*KRAS* c.183A>T *TSC2* c.4352G>A - -	*KRAS* c.183A>T *TSC2* c.4352G>A ***BRCA2* c.3586T>A** ***TSC2* c.4759T>C**
CC012	*KRAS* c.35G>A *TP53* c.839G>A	*KRAS* c.35G>A *TP53* c.839G>A
CC014	*TP53* c.818G>A - - -	*TP53* c.818G>A ***BRCA2* c.7573G>A** ***BRCA1* c.2905A>C** ***PDGFRA* c.1676G>T**
CC015	*BRCA1* c.2521C>T *TP53* c.524G>A - - -	*BRCA1* c.2521C>T *TP53* c.524G>A ***TSC1* c.1692A>T** ***PTCH1* c.2042C>A** ***PTCH1* c.29C>T**
CC016	*KRAS* c.35G>A *TP53* c.833C>T - -	*KRAS* c.35G>A *TP53* c.833C>T ***BRCA2* c.3961G>A** ***PIK3CA* c.1070G>T**
CC017	*KRAS* c.351A>C *TP53* c.844C>T	*KRAS* c.351A>C *TP53* c.844C>T
CC019	*KRAS* c.35G>A *TP53* c.778_799del - -	*KRAS* c.35G>A *TP53* c.778_779del ***BRCA1* c.2188G>A** ***PIK3CA* c.2983G>T**
CC020	*KRAS* c.182A>T *BRCA2* c.9440C>A *BRCA1* c.4039A>G *TP53*c.745A>G	*KRAS* c.182A>T *BRCA2* c.9440C>A *BRCA1* c.4039A>G *TP53*c.745A>G
CC021	*KRAS* c.34G>T *ARID1A* c.1833_1836del	*KRAS* c.34G>T *ARID1A* c.1833_1836del
CC022	*TP53* c.659A>G *ARID1A* c.2169G>C -	*TP53* c.659A>G *ARID1A* c.2169G>C ***BRCA2* c.5125G>A**
CC023	*KRAS* c.176C>G *KRAS* c.35G>A *TP53* c.641A>G	*KRAS* c.176C>G *KRAS* c.35G>A *TP53* c.641A>G
CC024	***TSC2* c.5383C>T** *NRAS* c.182A>G *ARID1A* c.3211delA	- *NRAS* c.182A>G *ARID1A* c.3216delA
CC025	*TP53* c.817C>T	*TP53* c.817C>T
CC026	*TP53* c.151G>T *ARID1A* c.1113delG	*TP53* c.151G>T *ARID1A* c.1113delG
CC027	*TP53* c.742C>T *PIK3CA* c.1633G>A	*TP53* c.742C>T *PIK3CA* c.1633G>A
CC028	*PTEN* c.445C>T *KRAS* c.38G>A *TSC2* c.5137C>T *BRCA1* c.3083G>A *TP53* c.817C>T *TP53* c.473G>A *ARID1A* c.4003C>T *PTCH1* c.1946G>A	*PTEN* c.445C>T *KRAS* c.38G>A *TSC2* c.5137C>T *BRCA1* c.3083G>A *TP53* c.817C>T *TP53* c.473G>A *ARID1A* c.4003C>T *PTCH1* c.1946G>A
CC029	*BRCA2* c.9004G>A *TP53* c.527G>A	*BRCA2* c.9004G>A *TP53* c.527G>A
CC030	*TP53* c.584T>C	*TP53* c.584T>C
CC031	*TP53* c.524G>A *ARID1A* c.5693C>T -	*TP53* c.524G>A *ARID1A* c.5693C>T ***KRAS* c.35G>T**
CC032	*NRAS* c.35G>A *PTCH1* c.3727G>A	*NRAS* c.35G>A *PTCH1* c.3727G>A
**Total # of variants**	**63**	**77**

Variants only detected by each laboratory are depicted in bold.

**Table 2:** Actionable variants identified by TumorSec^TM^ bioinformatics pipeline after sequencing of CRC FFPE samples, processed by two different library preparation protocols.

Sample	Capture-Based	Amplicon-Based
CC001	*TP53* c.524G>A	*TP53* c.524G>A
CC002	*KRAS* c.34G>T	*KRAS* c.34G>T
CC003	*KRAS* c.35G>T *TP53* c.524G>A	*KRAS* c.35G>T *TP53* c.524G>A
CC004	*PIK3CA* c.1634A>G *KRAS* c.183A>C	*PIK3CA* c.1634A>G *KRAS* c.183A>C
CC005	*TP53* c.455C>T	*TP53* c.455C>T
CC006	*KRAS* c.35G>A	*KRAS* c.35G>A
CC007	No variants detected	
CC009	No variants detected	***KRAS* c.182A>T** ***TP53* c.745A>G**
CC011	*KRAS* c.183A>T	*KRAS* c.183A>T
CC012	*KRAS* c.35G>A *TP53* c.839G>A	*KRAS* c.35G>A *TP53* c.839G>A
CC014	***TP53* c.818G>A** ***PDGFRA* c.1676G>T**	No variants detected
CC016	*KRAS* c.35G>A *TP53* c.833C>T ***PIK3CA* c.1070G>T**	*KRAS* c.35G>A *TP53* c.833C>T -
CC017	*KRAS* c.315A>C *TP53* c.844C>T	*KRAS* c.315A>C *TP53* c.844C>T
CC018	*KRAS* c.37G>T ***PTEN* c.607A>G**	*KRAS* c.37G>T -
CC019	*KRAS* c.35G>A ***TP53* c.778_779delTC** ***PIK3CA* c.2983G>T**	*KRAS* c.35G>A - -
CC021	*KRAS* c.34G>T	*KRAS* c.34G>T
CC022	*TP53* c.659A>G	*TP53* c.659A>G
CC023	*KRAS* c.35G>A *KRAS* c.176C>G *TP53* c.641A>G	*KRAS* c.35G>A *KRAS* c.176C>G *TP53* c.641A>G
CC024	*NRAS* c.182A>G	*NRAS* c.182A>G
CC025	*TP53* c.817C>T	*TP53* c.817C>T
CC026	***TP53* c.151G>T**	No variants detected
CC027	*PIK3CA* c.1633G>A *TP53* c.742C>T	*PIK3CA* c.1633G>A *TP53* c.742C>T
CC028	*KRAS* c.38G>A *TP53* c.473G>A *TP53* c.817C>T ***PTEN* c.445C>T**	*KRAS* c.38G>A *TP53* c.473G>A *TP53* c.817C>T -
CC029	*TP53* c.527G>A	*TP53* c.527G>A
CC030	*TP53* c.584T>C	*TP53* c.584T>C
CC031	*KRAS* c.35G>T *TP53* c.524G>A	*KRAS* c.35G>T *TP53* c.524G>A
CC032	*NRAS* c.35G>A -	*NRAS* c.35G>A ***TP53* c.376-1G>A**
11438	*TP53* c.472C>G	*TP53* c.472C>G
11442	No variants detected	
11545	*PIK3CA* c.1633G>A *KRAS* c.38G>A *KRAS* c.68T>G *TP53* c.481G>A ***PTEN* c.1-519T>C** ***TP53* c.911C>T** ***TP53* c.53C>T** ***EGFR* c.844G>A** ***EGFR* c.2264C>T**	*PIK3CA* c.1633G>A *KRAS* c.38G>A *KRAS* c.68T>G *TP53* c.481G>A - - - - -
11546	*TP53* c.743G>A	*TP53* c.743G>A
11548	*BRAF* c.1799T>A *PTEN* c.515G>A ***KRAS* c.118T>A**	*BRAF* c.1799T>A *PTEN* c.515G>A -
11557	*KRAS* c.34G>T	*KRAS* c.34G>T
11676	*TP53* c.843_862dup ***PIK3CA* c.2309G>A**	*TP53* c.843_862dup -
11678	*KRAS* c.35G>A ***PIK3CA* c.328_330del** ***EGFR* c.2314C>T**	*KRAS* c.35G>A - -
11681	*KRAS* c.436G>A *BRAF* c.1781A>G	*KRAS* c.436G>A *BRAF* c.1781A>G
11685	- *KRAS* c.38G>A *TP53* c.584T>A	***PIK3CA* c.1624G>A** *KRAS* c.38G>A *TP53* c.584T>A
11690	*PIK3CA* c.1258T>C *PTEN* c.800del	*PIK3CA* c.1258T>C *PTEN* c.800del
11691	*TP53* c.722C>G	*TP53* c.722C>G
11694	No variants detected	
117107	*KRAS* c.35G>T *PIK3CA* c.337_339del	*KRAS* c.35G>T *PIK3CA* c.337_339del
117118	*KRAS* c.35G>T	*KRAS* c.35G>T
117125	*KRAS* c.35G>A *TP53* c.916C>T *MET* c.2962C>T	*KRAS* c.35G>A *TP53* c.916C>T *MET* c.2962C>T
117134	No variants detected	
118142	*NRAS* c.182A>G ***PIK3CA* c.40C>A** ***PIK3CA* c.42C>G** ***PIK3CA* c.44T>G**	*NRAS* c.182A>G - - -
118144	*TP53* c.614A>G ***KRAS* c.187G>A** ***BRAF* c.1857G>C**	*TP53* c.614A>G - -
118159	*TP53* c.818G>C	*TP53* c.818G>C

**Table 3:** Actionable variants identified in samples processed with the amplicon-based AmpliSeq Cancer Hotspot Panel v2 but analyzed with different bioinformatics pipelines and compared with variants found using the capture-based library preparation and analyzed by TumorSec^TM^ bioinformatics.

Sample	Variant	TumorSec^TM^	CLC/ Franklin	LRM/ Franklin
11442	*KRAS* c.35G>T	✔	✔	✔
11545	*PIK3CA* c.1633G>A	✔	✔	✔
	*KRAS* c.38G>A	✔	✔	✔
	*KRAS* c.68T>G	✔	✔	✔
	*TP53* c.743G>A	✔	✔	✔
11548	*BRAF* c.1799T>A	✔	✔	✔
	*PTEN* c.515G>A	✔	✔	✔
11557	*KRAS* c.34G>T	✔	✔	✔
11676	*TP53* c.843_862dup	✔	✔	✔
11678	*KRAS* c.35G>A	✔	✔	✔
	*PIK3CA* c.328_330del	**✕**	✔	✔
11681	*KRAS* c.436G>A	✔	✔	✔
	*BRAF* c.1781A>G	✔	✔	✔
11685	*PIK3CA* c.1624G>A	✔	✔	✔
	*KRAS* c.38G>A	✔	✔	✔
	*TP53* c.584T>A	✔	✔	✔
11690	*PIK3CA* c.1258T>C	✔	✔	✔
	*PTEN* c.800del	✔	✔	✔
11691	*TP53* c.722C>G	✔	✔	✔
11694	No variants detected			
117107	*KRAS* c.35G>T	✔	✔	✔
	*PIK3CA* c.337_339del	✔	✔	✔
117118	*KRAS* c.35G>T	✔	✔	✔
117125	*KRAS* c.35G>A	✔	✔	✔
	*TP53* c.916C>T	✔	✔	✔
117134	No variants detected			
118142	*NRAS* c.182A>G	✔	✔	✔
	*TP53* c.614A>G	✔	✔	✔
118159	No variants detected			
CC001	*TP53* c.524G>A	✔	✔	✔
CC002	*KRAS* c.34G>C	✔	✔	✔
CC003	*KRAS* c.35G>T	✔	✔	✔
	*TP53* c.524G>A	✔	✔	✔
CC004	*KRAS* c.183A>C	✔	✔	✔
	*PIK3CA* c.1634A>G	✔	✔	✔
CC005	*TP53* c.455C>T	✔	✔	✔
CC006	*KRAS* c.35G>A	✔	✔	✔
CC007	No variants detected			
CC009	*PIK3CA* c.3140A>G	**✕**	**✕**	**✕**
CC011	*KRAS* c.183A>T	✔	✔	✔
CC012	*KRAS* c.35G>A	✔	✔	✔
	*TP53* c.839G>A	✔	✔	✔
CC014	*TP53* c.422G>A	**✕**	✔	✔
	*PDGFRA* c.1676G>T	**✕**	**✕**	**✕**
CC016	*KRAS* c.35G>A	✔	✔	✔
	*TP53* c.833C>T	✔	✔	✔
	*PIK3CA* c.1070G>T	**✕**	**✕**	**✕**
CC017	*KRAS* c.351A>C	✔	✔	✔
	*TP53* c.844C>T	✔	✔	✔
CC018	*PTEN* c.607A>G	**✕**	**✕**	**✕**
	*KRAS* c.37G>T	✔	✔	✔
CC019	*KRAS* c.35G>A	✔	✔	✔
	*TP53* c.382_383del	**✕**	**✕**	**✕**
	*PIK3CA* c.2983G>T	**✕**	**✕**	**✕**
CC021	*KRAS* c.34G>T	✔	✔	✔
CC022	*TP53* c.659A>G	✔	✔	✔
CC023	*KRAS* c.176C>G	✔	✔	✔
	*KRAS* c.35G>A	✔	✔	✔
	*TP53* c.245A>G	✔	✔	✔
CC024	*NRAS* c.182A>G	✔	✔	✔
CC025	*TP53* c.817C>T	✔	✔	✔
CC026	*TP53* c.34G>T	**✕**	**✕**	**✕**
CC027	*TP53* c.742C>T	✔	✔	✔
	*PIK3CA* c.1633G>A	✔	✔	✔
CC028	*PTEN* c.445C>T	**✕**	**✕**	**✕**
	*KRAS* c.38G>A	✔	✔	✔
	*TP53* c.817C>T	✔	✔	✔
	*TP53* c.473G>A	✔	✔	✔
CC029	*TP53* c.527G>A	✔	✔	✔
CC030	*TP53* c.584T>C	✔	✔	✔
CC031	*KRAS* c.35G>T	✔	✔	✔
	*TP53* c.524G>A	✔	✔	✔
CC032	*NRAS* c.35G>A	✔	✔	✔

## Data Availability

All NGS data generated can be requested directly from the authors.

## Supplementary Materials

The following supporting information can be downloaded at: https://www.mdpi.com/article/10.3390/cimb47080599/s1.

## Abbreviations

The following abbreviations are used in this manuscript:

BC	breast cancer
BTUCH	Biobank from the Universidad de Chile
CGL	Cancer Genomics Laboratory
CRC	colorectal cancer
DNA	deoxyribonucleic acid
FFPE	formalin-fixed, paraffin-embedded
GC	gastric cancer
GLOBOCAN	Global Cancer Observatory
IARC	International Agency for Research on Cancer
LC	lung cancer
NGS	next-generation sequencing
PC	prostate cancer
PCR	polymerase chain reaction
PVDs	Population Variant’s Databases
QC	quality control
ROIs	regions of interest
SNVs	single-nucleotide variants
TBLC	trachea–bronchus and lung
TCGA	The Cancer Genome Atlas
UK	United Kingdom
USA	United States of America
VAF	variant allele frequency
VCF	variant call format
