# Supplementary material for: Towards Personalized Precision Oncology: A Feasibility Study of NGS-Based Variant Analysis of FFPE CRC Samples in a Chilean Public Health System Laboratory
Source: Curr Issues Mol Biol. 2025 Jul 30;47(8):599. doi: 10.3390/cimb47080599 (PMC12384127; doi:10.3390/cimb47080599)
Supplement: Supplementary file 1 [file cimb-47-00599-s001.zip › Supplementary Table S1 - demographics.pdf]

Supplementary Table S1. FFPE samples patients' demographics

**Gender (number)**	
Male	50.7% (34)
Female	43.3% (29)
Unspecified	6.0% (4)
**Age**	
Male	61.2 y (33-85)
Female	60.6 y (45-86)
**Tumor histology**	
Tubular adenocarcinoma	74.6%
Mucinous adenocarcinoma	9.0%
Signet ring cell adenocarcinoma	4.5%
Unspecified	7.5%
Unknown	4.4%
**TNM**	
**T stage**	
T1	3.0% (2)
T2	11.9% (8)
T3	52.2% (35)
T4	19.4% (13)
Unknown	13.5% (9)
**N stage**	
N0	46.3% (31)
N1	22.3% (15)
N2	17.9% (12)
Unknown	13.5% (9)
**M stage**	
M0	0% (0)
M1	
M1a	17.9% (12)
M1b	9.0% (6)
M1c	17.9% (12)
Not specified	3.0% (2)
Unknown	52.2% (35)
