# Supplementary material for: Towards Personalized Precision Oncology: A Feasibility Study of NGS-Based Variant Analysis of FFPE CRC Samples in a Chilean Public Health System Laboratory
Source: Curr Issues Mol Biol. 2025 Jul 30;47(8):599. doi: 10.3390/cimb47080599 (PMC12384127; doi:10.3390/cimb47080599)
Supplement: Supplementary file 1 [file cimb-47-00599-s001.zip › Supplementary Table S2 - common genes.pdf]

**Supplementary Table S2.** List of genes analyzed by TumorSec$^{TM}$ and AmpliSeq v2 Cancer Hotspot kits. The genes shared by both assays are highlighted.

| **AmpliSeq Hotspot v2 panel (Illumina - 50 genes)** | **TumorSec$^{TM}$ panel (Salvo, M. et al., 2021 - 25 genes)** |
|---|---|
| ABL1 | AKT1 |
| AKT1 | ALK |
| ALK | ARID1A |
| APC | BRAF |
| ATM | BRCA1 |
| BRAF | BRCA2 |
| CDH1 | CDK4 |
| CDKN2A | EGFR |
| CSF1R | ERBB2 |
| CTNNB1 | ESR1 |
| EGFR | IDH2 |
| ERBB2 | KIT |
| ERBB4 | KRAS |
| EZH2 | MET |
| FBXW7 | MTOR |
| FGFR1 | NRAS |
| FGFR2 | PDGFRA |
| FGFR3 | PIK3CA |
| FLT3 | PTCH1 |
| GNA11 | PTEN |
| GNAQ | ROS1 |
| GNAS | SMO |
| HNF1A | TP53 |
| HRAS | TSC1 |
| IDH1 | TSC2 |
| IDH2 | |
| JAK2 | |
| JAK3 | |
| KDR | |
| KIT | |
| KRAS | |
| MET | |
| MLH1 | |
| MPL | |
| NOTCH1 | |
| NPM1 | |

| |
|---|
| NRAS |
| PDGFRA |
| PIK3CA |
| PTEN |
| PTPN11 |
| RB1 |
| RET |
| SMAD4 |
| SMARCB1 |
| SMO |
| SRC |
| STK11 |
| TP53 |
| VHL |
