# Supplementary material for: Towards Personalized Precision Oncology: A Feasibility Study of NGS-Based Variant Analysis of FFPE CRC Samples in a Chilean Public Health System Laboratory
Source: Curr Issues Mol Biol. 2025 Jul 30;47(8):599. doi: 10.3390/cimb47080599 (PMC12384127; doi:10.3390/cimb47080599)
Supplement: Supplementary file 1 [file cimb-47-00599-s001.zip › Supplementary Table S4 - VAF and actionability.pdf]

Supplementary Table S4. Summarizing table of actionable variants identified

Sample	Variant	Aminoacid change	VAF	Oncogenicity (somatic)	Actionability	Actionability in CRC
CC001	*TP53* c.524G>A	p.R175H	0.82	Oncogenic	Px1	Not listed
CC002	*KRAS* c.34G>T	p.G12C	0.44	Oncogenic	L1, R1, Dx2, FDA L2	L1, R1, FDA L2
CC003	*KRAS* c.35G>T	p.G12V	0.38	Oncogenic	L2, R1, Dx2, FDA L2	L4, R1, FDA L2
	TP53 c.524G>A	p.R175H	0.53	Oncogenic	Px1	Not listed
CC004	*PIK3CA* c.1634A>G	p.E545G	0.22	Oncogenic	L1, FDA L2	L3B, FDA L3
	KRAS c.183A>C	p.Q61H	0.39	Oncogenic	L2, R1, Dx2, FDA L2	L4, R1, FDA L2
CC005	*TP53* c.455C>T	p.P152L	0.34	Likely oncogenic	Px1	Not listed
CC006	*KRAS* c.35G>A	p.G12D	0.28	Oncogenic	L2, R1, Dx2, FDA L2	L4, R1, FDA L2
CC007	No variants	-	-	-	-	-
CC009	*KRAS* c.182A>T	p.Q61L	0.20	Oncogenic	L2, R1, Dx2, FDA L2	L4, R1, FDA L2
	TP53 c.745A>G	p.R249G	0.14	Likely oncogenic	Px1	Not listed
CC011	*KRAS* c.183A>T	p.Q61H	0.34	Oncogenic	L2, R1, Dx2, FDA L2	L4, R1, FDA L2
CC012	*KRAS* c.35G>A	p.G12D	0.32	Oncogenic	L2, R1, Dx2, FDA L2	L4, R1, FDA L2
	TP53 c.839G>A	p.R280K	0.45	Likely oncogenic	Px1	Not listed
CC014	*TP53* c.818G>A	p.R273H	0.15	Oncogenic	Px1	Not listed
	PDGFRA c.1676G>T	p.W559L	0.06	Unknown	-	-
CC016	*KRAS* c.35G>A	p.G12D	0.20	Oncogenic	L2, R1, Dx2, FDA L2	L4, R1, FDA L2
	TP53 c.833C>T	p.P278L	0.18	Likely oncogenic	Px1	Not listed
	PIK3CA c.1070G>T	p.R357L	0.05	Likely oncogenic	L1, FDA L2	L3B, FDA L3
CC017	*KRAS* c.351A>C	p.K117N	0.22	Oncogenic	L2, R1, Dx2, FDA L2	L4, R1, FDA L2
	TP53 c.844C>T	p.R282W	0.23	Likely Oncogenic	Px1	Not listed
CC018	*KRAS* c.37G>T	p.G13C	0.23	Oncogenic	L2, R1, Dx2, FDA L2	L4, R1, FDA L2
	PTEN c.607A>G	p.I203V	0.05	Unknown	-	-
CC019	*KRAS* c.35G>A	p.G12D	0.18	Oncogenic	L2, R1, Dx2, FDA L2	L4, R1, FDA L2
	TP53 c.778_779delTC	p.S260Qfs*3	0.22	Likely oncogenic	Px1	Not listed
	PIK3CA c.2983G>T	p.A995S	0.06	Unknown	-	-
CC021	*KRAS* c.34G>T	p.G12C	0.25	Oncogenic	L1, R1, Dx2, FDA L2	L1, R1, FDA L2
CC022	*TP53* c.659A>G	p.Y220C	0.19	Oncogenic	L3A, Px1, FDA L3	L3A, FDA L3
CC023	*KRAS* c.35G>A	p.G12D	0.22	Oncogenic	L2, R1, Dx2, FDA L2	L4, R1, FDA L2
	KRAS c.176C>G	p.A59G	0.19	Oncogenic	L2, R1, Dx2, FDA L2	L4, R1, FDA L2
	TP53 c.641A>G	p.H214R	0.33	Likely oncogenic	Px1	Not listed
CC024	*NRAS* c.182A>G	p.Q61R	0.06	Oncogenic	L2, R1, Dx2, Px1	L3B, R1, FDA L2
CC025	*TP53* c.817C>T	p.R273C	0.48	Likely oncogenic	Px1	Not listed
CC026	*TP53* c.151G>T	p.E51*	0.67	Likely oncogenic	Px1	Not listed
CC027	*PIK3CA* c.1633G>A	p.E545K	0.29	Oncogenic	L1, FDA L2	L3B, FDA L3
	TP53 c.742C>T	p.R248W	0.58	Likely oncogenic	Px1	Not listed
CC028	*KRAS* c.38G>A	p.G13D	0.55	Oncogenic	L2, R1, Dx2, FDA L2	L4, R1, FDA L2
	TP53 c.473G>A	p.R158H	0.39	Likely oncogenic	Px1	Not listed
	TP53 c.817C>T	p.R273C	0.38	Likely oncogenic	Px1	Not listed
	PTEN c.445C>T	p.Q149*	0.06	Likely oncogenic	L1, Dx3, FDA L2	L3B, FDA L3
CC029	*TP53* c.527G>A	p.C176Y	0.31	Likely oncogenic	Px1	Not listed
CC030	*TP53* c.584T>C	p.I195T	0.50	Likely oncogenic	Px1	Not listed
CC031	*KRAS* c.35G>T	p.G12V	0.06	Oncogenic	L2, R1, Dx2, FDA L2	L4, R1, FDA L2
	TP53 c.524G>A	p.R175H	0.15	Oncogenic	Px1	Not listed
CC032	*NRAS* c.35G>A	p.G12D	0.33	Oncogenic	L2, R1, Dx2, Px1	L3B, R1, FDA L2
	TP53 c.376-1G>A	NA	0.42	Likely oncogenic	Px1	Not listed

11438	*TP53* c.472C>G	p.R158G	0.41	Likely oncogenic	Px1	Not listed
11442	No variants	-	-	-	-	-
11545	*PIK3CA* c.1633G>A	p.E545K	0.33	Oncogenic	L1, FDA L2	L3B, FDA L3
	KRAS c.38G>A	p.G13D	0.22	Oncogenic	L2, R1, Dx2, FDA L2	L4, R1, FDA L2
	KRAS c.68T>G	p.L23R	0.27	Unknown	-	-
	TP53 c.481G>A	p.A161T	0.18	Likely oncogenic	Px1	Not listed
	PTEN c.1-519T>C	NA	0.06	Unknown	-	-
	TP53 c.911C>T	p.T304I	0.05	Unknown	-	-
	TP53 c.53C>T	p.T18I	0.05	Unknown	-	-
	EGFR c.844G>A	p.E282L	0.22	Unknown	-	-
	EGFR c.2264C>T	p.A755V	0.14	Unknown	-	-
11546	*TP53* c.743G>A	p.R248Q	0.32	Likely oncogenic	Px1	Not listed
11548	*BRAF* c.1799T>A	p.V600D	0.31	Oncogenic	L1, Dx2, FDA L2	L3B, FDA L3
	PTEN c.515G>A	p.R172K	0.06	Unknown	-	-
	KRAS c.118T>A	p.Y40N	0.09	Likely oncogenic	L2, R1, Dx2	L4, R1, FDA L2
11557	*KRAS* c.34G>T	p.G12C	0.51	Oncogenic	L1, R1, Dx2, FDA L2	L1, R1, FDA L2
11676	*TP53* c.843_862dup	p.N288Tfs*64	0.12	Likely oncogenic	Px1	Not listed
	PIK3CA c.2309G>A	p.R770Q	0.29	Unknown	-	-
11678	*KRAS* c.35G>A	p.G12D	0.21	Oncogenic	L2, R1, Dx2, FDA L2	L4, R1, FDA L2
	PIK3CA c.328_330del	p.E110del	0.22	Oncogenic	L1, FDA L2	L3B, FDA L3
	EGFR c.2314C>T	p.P772S	0.11	Likely oncogenic	Unknown	Not listed
11681	*KRAS* c.436G>A	p.A146T	0.31	Oncogenic	L2, R1, Dx2, FDA L2	L4, R1, FDA L2
	BRAF c.1781A>G	p.D594G	0.28	Oncogenic	L2, Dx3, FDA L3	Not listed
11685	*PIK3CA* c.1624G>A	p.E542K	0.09	Oncogenic	L1, FDA L2	L3B, FDA L3
	KRAS c.38G>A	p.G13D	0.17	Oncogenic	L2, R1, Dx2, FDA L2	L4, R1, FDA L2
	TP53 c.584T>A	p.I195N	0.16	Likely oncogenic	Px1	Not listed
11690	*PIK3CA* c.1258T>C	p.C420R	0.18	Oncogenic	L1, FDA L2	L3B, FDA L3
	PTEN c.800del	p.K267Rfs*9	0.31	Likely oncogenic	L1, Dx3, FDA L2	L3B, FDA L3
11691	*TP53* c.722C>G	p.S241C	0.42	Likely oncogenic	Px1	Not listed
11694	No variants	-	-	-	-	-
117107	*KRAS* c.35G>T	p.G12V	0.48	Oncogenic	L2, R1, Dx2, FDA L2	L4, R1, FDA L2
	PIK3CA c.337_339del	p.L113del	0.23	Likely oncogenic	L1, FDA L2	L3B, FDA L3
117118	*KRAS* c.35G>T	p.G12V	0.13	Oncogenic	L2, R1, Dx2, FDA L2	L4, R1, FDA L2
117125	*KRAS* c.35G>A	p.G12D	0.24	Oncogenic	L2, R1, Dx2, FDA L2	L4, R1, FDA L2
	TP53 c.916C>T	p.R306*	0.58	Likely oncogenic	Px1	Not listed
	MET c.2962C>T	p.R970C	0.52	Inconclusive	-	-
117134	No variants	-	-	-	-	-
118142	*NRAS* c.182A>G	p.Q61R	0.40	Oncogenic	L2, R1, Dx2, Px1	L3B, R1, FDA L2
	PIK3CA c.40C>A	p.H14N	0.06	Unknown	-	-
	PIK3CA c.42C>G	p.H14Q	0.06	Unknown	-	-
	PIK3CA c.44T>G	p.L15W	0.06	Unknown	-	-
118144	*TP53* c.614A>G	p.Y205C	0.44	Likely oncogenic	Px1	Not listed
	KRAS c.187G>A	p.E63K	0.10	Likely oncogenic	L2, R1, Dx2	L4, R1, FDA L2
	BRAF c.1857G>C	p.W619C	0.08	Unknown	-	-
118159	*TP53* c.818G>C	p.R273P	0.39	Likely oncogenic	Px1	Not listed

- VAF stands for Variant Allele Frequency
- Actionability according with OncoKB criteria
- Not listed in last column means that, according with OncoKB database, there is no FDA-approved or NCCN-compendium listed treatments specifically for CRC patients harboring that particular mutation.
- NA stands for Not Applicable
